# Editorial: Cognitive rehabilitation: a multidisciplinary approach

**DOI:** 10.3389/fresc.2023.1268531

**Published:** 2023-09-04

**Authors:** Hanneke E. Hulst, Ekaterina Dobryakova, Silvana L. Costa, Sarah J. Donkers

**Affiliations:** ^1^ Health-, Medical-, and Neuropsychology Unit, Institute of Psychology, Leiden University, Leiden, Netherlands; ^2^ Center for Traumatic Brain Injury Research, Kessler Foundation, East Hanover, NJ, United States; ^3^ Center for Neuropsychology and Neuroscience Research, Kessler Foundation, East Hanover NJ, United States; ^4^ School of Rehabilitation Science, College of Medicine, University of Saskatchewan, Saskatoon, SK, Canada

**Keywords:** cognitive rehabilitation, cognitive assessment, multidisciplinary approach, stroke, ageing, multiple sclerosis

**Editorial on the Research Topic** Cognitive rehabilitation: a multidisciplinary approach

Studies on cognition and cognitive rehabilitation are complex due to the intricate nature of human cognition and its impact on various aspects of life. For example, cognitive impairment has far-reaching effects on an individual's daily functioning, emotional well-being, social interactions and overall quality of life (1).

For many people with neurocognitive disorders [e.g., multiple sclerosis (MS), Parkinson's disease, acquired brain damage] having adequate cognitive functioning is not always self-evident, hampering life in many domains (2–4). The urgency for scientists and clinicians to come up with successful cognitive rehabilitation interventions is clearly felt. It is this urgency that has led to the creation of a Research Topic in Frontiers in Rehabilitation Sciences titled “Cognitive Rehabilitation: a multidisciplinary approach”. This Research Topic brings together research and expertise from several disciplines, such as neuroscience, psychology, and occupational therapy, to explore innovative approaches to cognitive rehabilitation. Six articles have been included in this Research Topic, emphasizing the benefits of a multidisciplinary approach as it allows for a comprehensive understanding of the complex nature of neurocognitive disorders and hopefully will lead to the development of more effective and tailored interventions.

To date, we are still in need of a better understanding of the underlying biological mechanisms (such as brain functioning) of complex processes contributing to cognitive function and behavioral change. Additionally, we need more effective interventions that can be implemented into clinical practice (5, 6). Despite efforts over the past decades, e.g., making cognitive rehabilitation a discipline on its own and running a variety of clinical trials, we are not there yet. The multiple attempts introducing new interventions, both pharmacological as well as non-pharmacological (7), for individuals with MS for example, have brought us to the following position: cognitive rehabilitation is, on group level, beneficial to persons with neurocognitive disorders. However, we struggle with several methodological issues, such as (in)sufficient sample sizes, ecological validity, and heterogeneity in treatment response amongst persons, which has been impeding implementation in clinical practice. In the current Research Topic, Nancy Chiaravalloti and Aubree Alexander make an argument for better collaboration between scientists and clinicians or, to use their own words, “for more flexibility around evidence-based protocols to answer to the more fluid demands of the treatment environment” [*Strengthening the connection between clinical research and clinical practice of cognitive rehabilitation*]. A better integration between scientists and clinicians, but also between the different perspectives of all experts involved, is indeed what we urgently need.

Cognitive rehabilitation is about cognitive functions that has been lost. It is however also about wishes and values of the person suffering from the cognitive deficits and the external world the person is living in, such as work, home, and social settings. As such, the primary goal of cognitive rehabilitation is dependent on the perspective of the expert. The neuropsychologist is most likely primarily focused on enhancement of cognitive functioning, i.e., is there a significant improvement on objective measures of cognition before and after cognitive rehabilitation? For an occupational therapist, the significant improvement on neuropsychological tests in itself would not be sufficient. Almost independent of the underlying diagnosis, occupational therapists would be more concerned with the person's instrumental activities of daily life. One such activity is fitness to drive. Instead of using a paper-and-pencil cognitive test, Rosenfeld and colleagues used a driving test for elderly people who were evaluated by a licensed driving instructor. Next, the researchers investigated whether self-awareness about driving performance prior to the driving test would predict the outcome of the evaluation. Curious about the results? They can be read in the current Research Topic [*Self-awareness predicts fitness to drive among adults referred to occupational therapy evaluation*].

Beyond the perspectives of the different experts involved in cognitive rehabilitation, many people with neurocognitive disorders struggle with comorbidities such as fatigue, mood problems and anxiety (8, 9). In scientific research projects, it is not uncommon to exclude individuals with (severe) comorbidities to ensure that the study's focus remains on the effectiveness of the specific interventions being tested. This approach allows for a clearer understanding of the intervention's direct impact and the targeted cognitive abilities. However, in real-world clinical practice, it is vital to acknowledge and address these comorbidities as they significantly impact the daily lives of individuals with neurocognitive disorders. There is still no holy grail to the treatment of fatigue. Therefore, we were happy to welcome the protocol paper of Lisa Walker and colleagues in which they present their study design for developing a behavioral intervention for cognitive fatigability in persons with MS [*Development of a behavioural intervention for cognitive fatigability in multiple sclerosis: Protocol for a pilot and feasibility study*]. It can be expected that a successful intervention for fatigue may significantly improve the quality of life of persons with MS. A more outside-the-box idea may be, that remedying fatigability or any of the abovementioned comorbidities before the start of cognitive rehabilitation, may actually enhance the effects of the cognitive rehabilitation afterwards, for example, by making more mind space for the patient to focus on the rehabilitation. However, whether a so-called pre-rehabilitation is adding to the effectiveness of interventions is something that needs further exploration.

Cognitive rehabilitation is about selecting the best suitable instruments to identify patients that are in need for cognitive rehabilitation. Consequently, in this Research Topic a Spanish consensus on cognitive assessment for people with MS is presented by Higueras and colleagues [*Cognitive assessment in patients with multiple sclerosis: A Spanish consensus*].

Additionally, cognitive rehabilitation is about exploring innovative interventions and about selecting the most-effective treatment protocols. In this Research Topic, two papers focused on the use of transcranial direct current stimulation [*tDCS over the left prefrontal Cortex improves mental flexibility and inhibition in geriatric inpatients with symptoms of depression or anxiety: A pilot randomized controlled trial*] and transcranial electrical stimulation [*Can Transcranial Electrical Stimulation Facilitate Post-stroke Cognitive Rehabilitation? A Systematic Review and Meta-Analysis*] as such innovative interventions for geriatric and post-stroke patients respectively. Results do show potential and need to be followed up in the near future.

Lastly, cognitive rehabilitation is about selecting outcome measures that are meaningful to clinicians and patients. It requires interdisciplinary collaboration between professionals in various fields that are open to new ideas and are willing to enrich their perspectives and accordingly change them. If, as a community, we can foster such a collaborative and open-minded environment, we will be able to make significant strides in improving the lives of individuals with neurocognitive disorders and enhance their overall well-being.
